# Polyphenolic HRMS Characterization, Contents, Antioxidant Activity and Cytotoxic Effects of Costa Rican *Ocimum tenuiflorum* L. Extracts

**DOI:** 10.3390/molecules31193546

**Published:** 2026-10-04

**Authors:** Andrea Mariela Araya Sibaja, Juan Diego Chacón-Vargas, Luis Felipe Vargas-Huertas, Gabriela Azofeifa, Silvia Quesada, José Roberto Vega-Baudrit, Andrés Sánchez-Kopper, Diego Alvarado-Corella, Andrea Monge-Navarro, Mirtha Navarro-Hoyos

**Affiliations:** 1 BIODESS, Department of Chemistry, University of Costa Rica, San Pedro de Montes de Oca 11501, Costa Rica; aaraya@cenat.ac.cr (A.M.A.S.); juan.chaconvargas@ucr.ac.cr (J.D.C.-V.); luis.vargashuertas@ucr.ac.cr (L.F.V.-H.); diegoallcore@gmail.com (D.A.-C.); andimongenavarro@gmail.com (A.M.-N.); 2 National Laboratory of Nanotechnology, CeNAT, Pavas, San José 10109, Costa Rica; jvegab@gmail.com; 3 R&D+i Department, INNOBIOTIQ SRL, Pavas, San José 10107, Costa Rica; 4 Department of Biochemistry, School of Medicine, University of Costa Rica, San Pedro de Montes de Oca 11501, Costa Rica; gabriela.azofeifacordero@ucr.ac.cr (G.A.); silvia.quesada@ucr.ac.cr (S.Q.); 5 Department of Chemistry, Chemistry and Microbiology Research and Services Center, Costa Rica Institute of Technology, Cartago 30109, Costa Rica; ansanchez@tec.ac.cr; 6 Chemistry Department, Georgetown University, Washington, DC 20037, USA

**Keywords:** *Ocimum tenuiflorum*, holy basil, polyphenols, antioxidant, cytotoxicity

## Abstract

Holy basil (*Ocimum tenuiflorum*) is well known for its bioactive properties, including antioxidant and anti-inflammatory effects. Leaves samples from Costa Rica were extracted and analyzed in composition and in antioxidant and cytotoxic activities. The analysis by Ultra Performance Liquid Chromatography coupled with High Resolution Mass Spectrometry using a quadrupole-time-of-flight analyzer (UPLC-QTOF) allowed the identification of 12 compounds, including 7 glycosylated flavonoids, 2 coumaroyl alcohols, rosmarinic acid, eugenol, and salvianolic acid E. Quantification using liquid chromatography with diode array detector (UPLC-DAD) was performed for the main components, including apigenin and luteolin glucoronides, rosmarinic acid, and eugenol, revealing an overall content of 78.28–121.05 mg/g dry extract. Total phenolic values ranged between 74.5 and 122.5 mg GAE/g dry extract, while antioxidant evaluations showed IC_50_ values of 28.18–48.18 mg dry extract/L for 2,2-diphenyl-1-picrylhidrazyl (DPPH) assay, 1192.8–2006.5 μmol Trolox/g dry extract for Ferric Reducing Antioxidant Power (FRAP), 2.56–3.01 μmol Trolox/mg dry extract for Oxygen Radical Absorbance Capacity (ORAC), and 708.7–1415.8 μmol Trolox/g dry extract for Trolox Equivalent Antioxidant Capacity (TEAC). Pearson correlation analyses (*p* < 0.05) between the quantified phenolic content and the antioxidant assays showed the highest values between UPLC and DPPH (R = 0.962) and UPLC with ORAC values (R = 0.955). A Relative Antioxidant Capacity Index (RACI) evaluation indicated HB-1 and HB-3 samples as the ones with the best results in these antioxidant assays, aligning with their higher content in polyphenols, which can be attributed to their higher maturity at the harvesting stage in comparison with HB-2. The in vitro cytotoxicity evaluation of these extracts in adenocarcinoma cell lines, using the 3-(4,5-dimethylthiazol-2-yl)-2,5-diphenyl-2H-tetrazolium bromide (MTT) assay, showed a significant cytotoxic effect against AGS gastric cancer cell lines with an IC_50_ of 78–150 μg extract/mL and a selectivity index greater than 6 in respect to normal Vero cell lines. To the best of our knowledge, this work represents the first study of its kind in *O. tenuiflorum* from Costa Rica.

## 1. Introduction

Holy basil (*Ocimum tenuiflorum*), also known as Tulsi, is a plant of the Lamiaceae family, long recognized in traditional medicine, whose properties have been described in the Indian medicinal text Ayurveda (The Science of Life), believed to be about 5000 years old [1]. Regarding the origin of this plant, analysis of molecular evolution suggests North-Central India as the geographical origin of this species that has extended throughout the eastern world tropics [2].

*O. tenuiflorum* is of special interest due to its flavor and fragrance and as a subject of early phytochemical and bioactivity studies [3]. Scientific exploration of the traditional medicinal claims of Tulsi gained momentum in the mid-20th century [1], and ethnomedicine surveys reveal *O. tenuiflorum* as a plant used to treat diverse ailments like cough and cold, malaria, and erectile dysfunction [4], as well as antifungal and analgesic activities [5,6].

Different studies have shown the antioxidant activity of *O. tenuiflorum* extracts. For instance, a study suggested their potential use as a preservative in foods and their modulatory properties in human biology under conditions of excessive oxidative stress [7]. Further, the antioxidant effects of leaf extracts were found to be at least partially responsible for the amelioration of axotomy-induced axonal degeneration and neuropathy in rats [8].

Antioxidant properties play a crucial role in anti-inflammatory, anti-diabetic, and anti-cancer properties. For instance, *O. tenuiflorum* has shown effects in stress-induced disorders and as an immunomodulator [9,10,11]. In addition, it has also exhibited promising hypoglycemic properties in induced-diabetic rats, with significant reduction in total cholesterol, triglycerides, and total lipids [12,13], as well as an increase in insulin release [14].

Anti-inflammatory effects were shown in studies in mice with carrageenan-induced paw inflammation, indicating that *O. tenuiflorum* extracts significantly reduced the induced edema and granuloma [15]. Further studies indicated that anti-inflammatory effects might be attributed to the inhibition of cyclooxygenase and lipo-oxygenase pathways on the arachidonic acid metabolism, based in structure-activity relationships of flavonoids, relative to COX-1 inhibitory activity [16].

Several studies have reported promising cytotoxicity activity of *O. tenuiflorum* extracts against cancer cell lines, particularly in vitro studies using MTT, which indicated an inhibitory effect of leaf extracts on gastric cancer AGS cell lines [17], as well as on NCI-H460 lung adenocarcinoma cell lines [18] and on AsPc-1 and MiaPaCa lung cancer cells [19]. Mechanistic studies using promising extracts have shown an increase of the apoptosis signaling via the mitochondrial intrinsic pathway and chemopreventive-induced gastric carcinogenesis [20,21].

These reported bioactivities are closely linked to the *O. tenuiflorum*-rich profile of secondary metabolites. Leading compounds identified in holy basil leaves include polyphenols and triterpene glycosides [22]. Polyphenols play a crucial role in a wide range of physiological and biochemical processes, which are dependent also on factors like geographical region, environmental and growth conditions [23].

Further, polyphenols reported in *O. tenuiflorum,* such as phenolic alcohols, phenolic acids, phenylpropanoids, and O-glycosylated flavonols, as well as glucoronides, O-glycosylated and C-glycosylated flavones, have shown a plethora of antioxidant and anti-inflammatory effects [24], for instance, controlling cellular stress response and exhibiting anticancer properties, as well as regulating apoptosis [25,26].

Hence, the aim of this work is to characterize the main polyphenolic metabolites in leaf extracts of *Ocimum tenuiflorum* cultivated in Costa Rica and to perform their quantification as well as the evaluation of their antioxidant capacity and cytotoxic activity on normal and different adenocarcinoma cell lines to assess their selectivity and bioactivity linked to potential health benefits.

## 2. Results and Discussion

### 2.1. HRMS Characterization

The UPLC-QTOF analyses of *O. tenuiflorum* extracts, described in the Materials and Methods section, allowed the identification of 12 phenolic compounds, which are summarized in Table 1. In turn, the extracted ion chromatograms (XIC) of these compounds are presented in Figure S1 in the Supplementary Materials.

**Figure 1:**
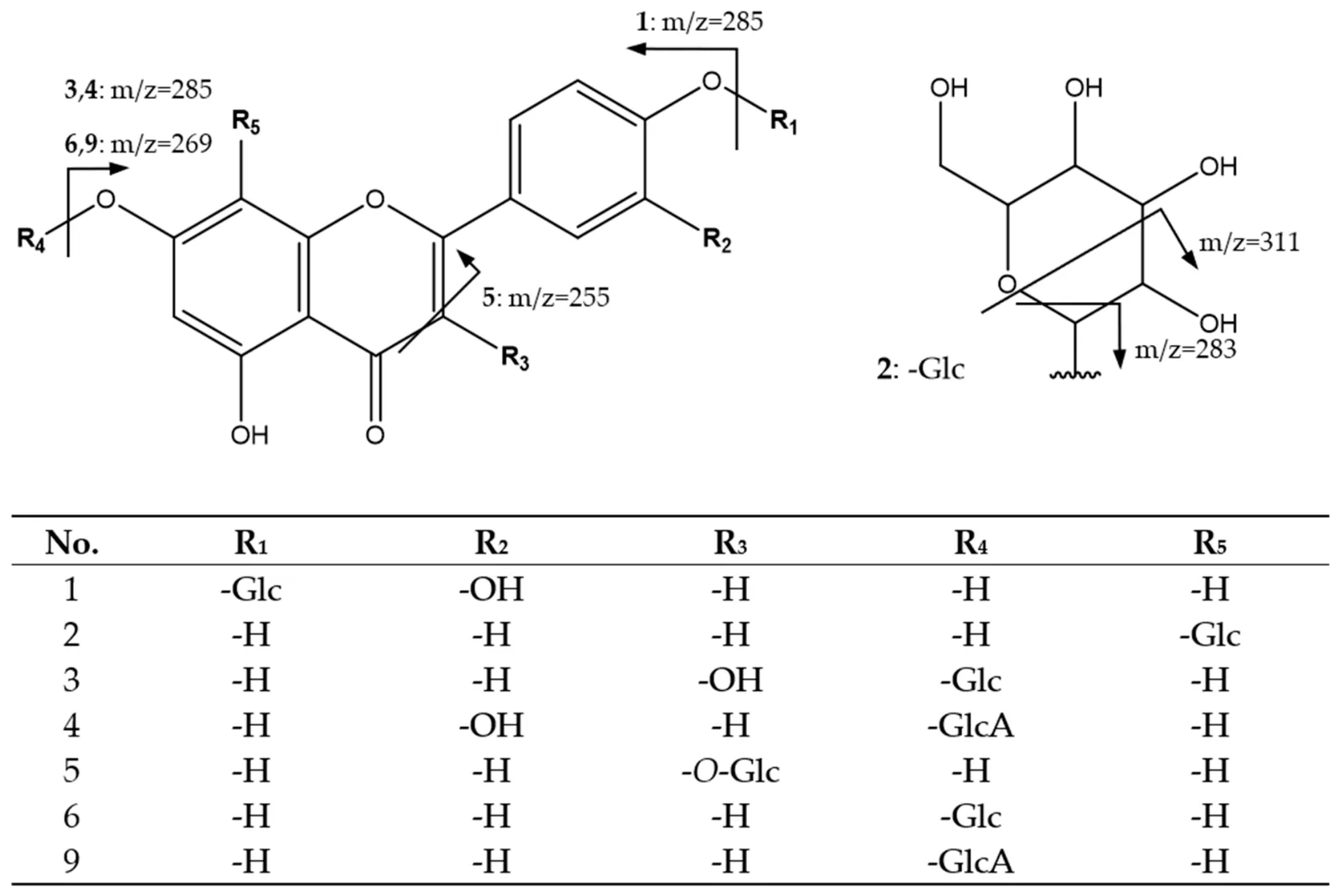
Structures and main fragmentation of compounds **1**, **2**, **3**, **4**, **5**, **6,** and **9**. Glc: glucoside, GlcA: glucuronic acid.

The first type of compounds present in the samples corresponds to glucoronides and glycosylated derivatives of flavonoids, namely peaks **1**, **2**, **3**, **4**, **5**, **6**, and **9**, whose structures and fragmentation patterns are represented in Figure 1.

Peaks **1** (18.75 min), **3** (21.65 min), and **5** (24.60 min) show a molecular ion [M-H]^−^ = 447.0926 Da (C_21_H_19_O_11_); however, they exhibit different fragmentation patterns. For instance, peak **1** has a main fragment at 285 Da [M-H-162]^−^, from the loss of the hexose and coincident with a luteolin moiety; thus, peak **1** was identified as luteolin-4′-*O*-glucoside [27,28].

Peak **3** shows main fragments at 285 Da and 284 Da from the loss of the hexose, while peak **5** has its main fragments at 284 Da and at 255 Da from the additional loss of CHO. Fragments at 284 Da and 285 Da indicate a kaempferol aglycone, while the fragment at 255 Da was previously reported specifically for a 3-*O*-glucoside [27]; thus, peak **3** is identified as kaempferol-7-*O*-glucoside and peak **5** as kaempferol-3-*O*-glucoside [27,28,29].

Peak **2** (20.28 min), [M-H]^−^ = 431.0980 Da (C_21_H_19_O_10_), is identified as C-glycoside vitexin, with characteristic fragments at 311 Da and 283 Da originated by the expected glycoside cleavages [27,30,31], as shown in Figure 1. Peak **4** at 23.09 min, [M-H]^−^ = 461.0721 Da (C_21_H_17_O_12_), corresponds to luteolin-7-*O*-glucuronide, with a main fragment at 285 Da [M-H-176]^−^ from the loss of the glucuronide moiety, which results in the luteolin aglycone [30].

In turn, peak **6** (25.26 min) has a [M-H]^−^ = 431.0980 Da (C_21_H_19_O_10_) and a main fragment at 269 Da [M-H-162]^−^, which corresponds to the apigenin aglycone moiety, after the loss of glucose as shown in Figure 1. Thus, this peak was assigned to apigenin-7-*O*-glucoside [10,27]. Finally, peak **9** (26.79 min) has a [M-H]^−^ = 445.0767 Da (C_21_H_17_O_11_), and it is assigned to apigenin-7-O-glucoronide due to its main fragment at 269 Da [M-H-176]^−^ that corresponds to the apigenin aglycone from the loss of the glucuronide [30,32], as shown in Figure 1.

**Figure 2:**
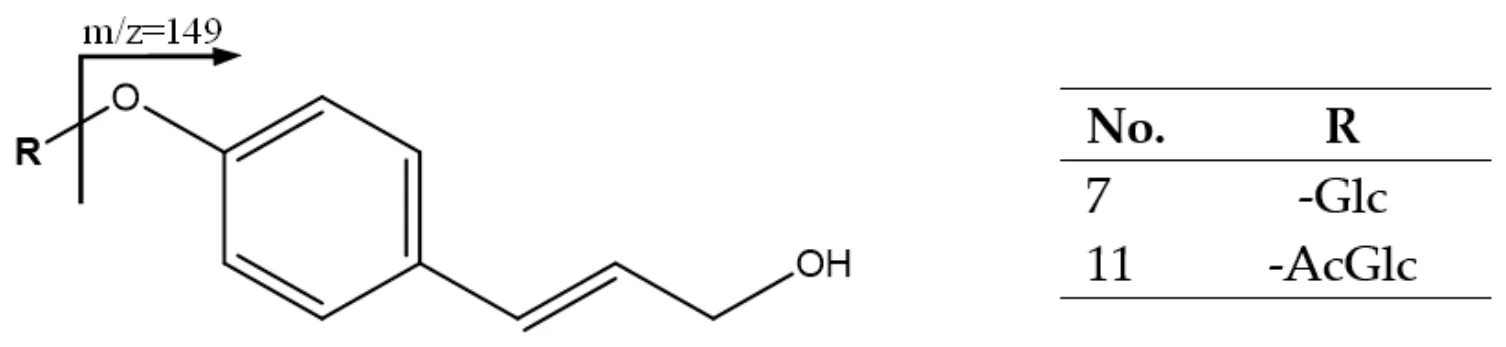
Structures and fragmentation of *p*-coumaryl alcohol glycosilated derivatives **7** and **11**. Glc: glucoside, AcGlc: acetylglucoside.

A second type of compound present in the samples corresponds to phenylpropanoid alcohols, namely peaks **7** and **11**, whose structures and fragmentation pattern are represented in Figure 2. In fact, Peak **7** (25.38 min) with [M-H]^−^ = 311.1129 Da (C_15_H_19_O_7_) was assigned to *p*-coumaryl alcohol 4-O-glucoside, whose fragmentation yields a main peak at 149 Da [M-H-162]^−^ due to the loss of the glucoside moiety [33].

In turn, peak **11** (29.91 min) shows a [M-H]^−^ = 353.1234 Da (C_17_H_21_O_8_) with main fragments at 149 Da indicating the presence of a *p*-coumaroyl alcohol aglycone and at 293 Da [M-H-60]^−^ suggesting the presence of an acetyl group. Thus, allowing this peak to be assigned to *p*-coumaryl alcohol 4-*O*-acetylglucoside [34,35,36].

Peak **12** (31.05 min) was identified as eugenol, with a visible [M-H]^−^ ion at 163.0755 Da corresponding to C_10_H_11_O_2_ and a main fragment at 148 Da that corresponds to the loss of the methyl from the methoxy group [37,38].

**Figure 3:**
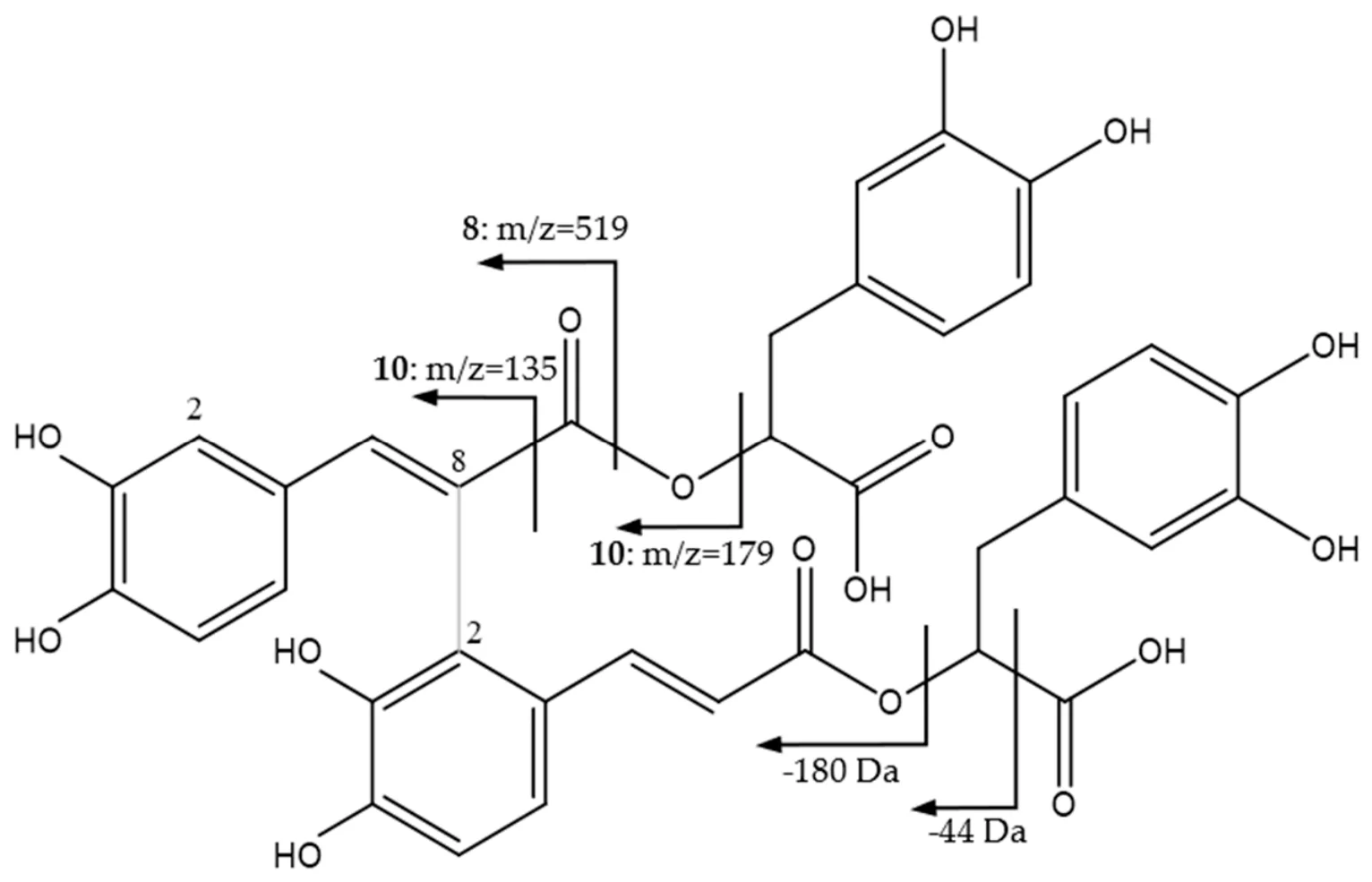
Structure and main fragments of salvianolic acid E **8**.

Finally, another type of compound present in the samples corresponds to rosmarinic acid **10** and its dimer **8**, whose structure and fragments are shown in Figure 3. In fact, peak **10**, at 27.73 min, shows a molecular ion [M-H]^−^ = 359.0766 Da (C_18_H_15_O_8_), with characteristic fragments at 179 Da from the caffeic acid moiety and 135 Da from additional loss of CO_2_ [30], thus assigned as rosmarinic acid.

Finally, peak **8** (25.59 min), with molecular ion [M-H]^−^ = 717.1447 Da (C_36_H_29_O_16_), was identified as salvianolic acid E, a dimer of rosmarinic acid with a *C*8→*C*2 bond, as shown in Figure 3. The main fragments correspond to 519 Da from the loss of a dihydroxyphenyllactic acid moiety, 475 Da (519 − 44 Da) from an additional loss of CO_2_, and 339 Da (519–180 Da) due to the loss of a second unit of dihydroxyphenylpropanoic acid [32,39,40].

### 2.2. UPLC-DAD Quantification

The quantification of phenolic compounds was performed by UPLC-DAD as described in the Materials and Methods section. Table 2 summarizes the quantification results for luteolin-7-O-glucoronide and apigenin-7-O-glucoronide, as well as for rosmarinic acid and eugenol. The corresponding chromatograms for the sample analyses are shown in Figure S2 in the Supplementary Materials.

The quantification by UPLC-DAD shows that HB-1 holds the highest content of phenolic compounds, followed closely by HB-3. In turn, HB-2 is the one with the lowest total content of polyphenols. The fact that HB-1 and HB-3 values are similarly higher can be attributed to the fact that these were harvested at ninety days of maturity, while HB-2 corresponded to sixty days. In fact, studies on harvesting stages in respect to the maturity of the leaves have shown higher phenolic content at later stages corresponding to polyphenol accumulation as a response to the plant growth stress with maturity, indicating also that the impact is dependent on species and cultivars [41,42].

The polyphenol content expressed by mass of dry weight, once calculations considering the extract yields are applied, follows the same trend as the one expressed per mass of extract, with HB-1 and HB-3 showing similar higher content of polyphenols, explained as mentioned earlier by their similar greater harvesting maturity in respect to HB-2 [41,42]. Regarding individual compounds, eugenol is the main one in all samples with a content of 47.74–86.49 mg/g extract and 2.710–4.388 mg/g DW.

Comparing with the literature, there is variability in the polyphenol content reported. For instance, studies of *O. tenuiflorum* leaf extracts from India have shown values of 6.58–10.0 mg/g extract [43] and 13.8–54.6 mg/g extract [44], lower than the ones reported in the present study (78.28–121.05 mg/g extract). In turn, other reports indicate a content between 0.03–20.7 mg/g FW [45] for leaves of plants cultivated in the USA and 0.79–2.2 mg/g DW [46] for leaves from India, compared to values in a similar range (4.44–6.14 mg/g DW) in this study. In respect to the main metabolites, the present results fit within the range of values reported for rosmarinic acid (0.8–6.7 mg/g DW) [7,43] and for eugenol (0.7–12 mg/g DW) [22,43] in *O. tenuiflorum* leaves from India. As it is discussed in the following sections, the variability of results fits an expected range in agreement with sample maturity and growth regions and conditions with relevant bioactivity results correlating with the different samples polyphenols content, as published earlier [41,42].

### 2.3. Total Phenolic Determination

The results for total phenolic content, following the Folin-Ciocalteau method as described in the Materials and Methods section, expressed per g of dry extract and per g of dry weight, are presented in Table 3.

The results shown in Table 3 for total phenolics (TP) values per gram of extract are consistent with the UPLC quantification, where values for samples HB-1 and HB-3 are similar and higher than those for HB-2, which can be attributed, as mentioned earlier, to this last sample harvested at an earlier maturity stage with less accumulation of polyphenols [41,42]. The results for total phenolics per gram of dry weight show the same trend, with sample HB-3 holding a slightly higher value than HB-1 due to the extraction yields.

In respect to the literature, the results obtained for the present extracts are similar to those reported for *O. tenuiflorum* leaf extracts from India, ranging from 87.13 to 124.3 mg GAE/g extract [30,47], and higher or similar than those reported for leaf extracts from Thailand [48,49] ranging between 50.2 and 115.6 mg GAE/g extract. In turn, results expressed per dry weight indicate the present extracts hold a lower comparative range than values for *O. tenuiflorum* leaves of plants cultivated in Pakistan [50] and Malaysia [51], ranging between 7–13.5 mg GAE/g DW and 11.47–18.15 mg GAE/g DW, respectively.

### 2.4. Antioxidant Activity Evaluation

The results for the antioxidant activity evaluations described in the Materials and Methods section, expressed per dry extract weight and per dry leaf weight, are summarized in Table 4.

HB-1 and HB-3 extracts show the best overall results with similar responses, while HB-2 shows around 15% lower values in ORAC, 40% in DPPH and FRAP analyses, and 50% lower values in TEAC. Results expressed per dry weight have a similar trend to the extract outcomes, with similar higher values for HB-1 and HB-3, while HB-2 remains the sample exhibiting the least antioxidant effect. These results can be attributed, as previously mentioned, to the HB-2 sample’s lower maturity at harvesting, thus holding lower content of polyphenols with potential antioxidant activity, while HB-1 and HB-3 correspond to leaves with higher maturity, holding higher polyphenolic content, thus greater antioxidant potential [41,42]. The results of each antioxidant assay are discussed in the following sections.

#### 2.4.1. DPPH Evaluation

The results obtained for all samples using the DPPH methodology are consistent with the total phenolic quantification, with HB-1 and HB-3 samples holding the best antioxidant activity in agreement with a higher content of polyphenols, and HB-2 showing a lesser antioxidant value analogous to its lower polyphenolic content [52].

Comparing with the literature, results for *O. tenuiflorum* extracts from Thailand [49] and India [53] indicate IC_50_ values ranging from 8.9 to 31.2 mg extract/L and 5.6 to 71.6 mg extract/L, respectively, indicating high antioxidant activity. In turn, other studies report lower antioxidant values with IC_50_ ranging between 340 and 630 mg extract/L for *O. tenuiflorum* leaves from India [7] and up to 720 mg extract/L for leaves from Pakistan [54]. The present results (28.2–48.2 mg extract/mL) fit within the range of the first group of reports with better antioxidant activity. Another report for *O. tenuiflorum* leaves from India [55] indicates 27.7–61.8% inhibition for 0.50–3.33 mg DW/L compared to 0.555–0.848 mg DW/L samples in the present study, achieving 50% inhibition, thus indicating a similar range of antioxidant activity.

#### 2.4.2. FRAP Evaluation

The results obtained with the FRAP methodology indicate again that HB-2 remains as the poorest sample, yielding a value 40% lower than HB-3. In respect to the literature, a study of *O. tenuiflorum* leaf extracts from Thailand [49] indicates values ranging between 62.4 and 147.6 mg TE/g extract compared with a higher range from 298 to 502 mg TE/g extract in the present work. Another study in *O. tenuifloruim* leaves from Thailand [56] indicates values between 48 and 244 μmol Trolox/g DW, with the present results (67.7–110.3 μmol Trolox/g DW) fitting within that range.

In turn, a study on *O. tenuiflorum* leaves from Malaysia [51] reported values of 98.4–601.9 μM Fe(II)/g DW, while another on leaves from India [57] indicated a value of 294.2 μmol Fe(II)/g DW, with the present study exhibiting similar results (149.2–248.3 μM Fe(II)/g DW).

#### 2.4.3. TEAC Evaluation

The results for the TEAC analysis also show that HB-3 yields the best antioxidant activity, followed closely by HB-1, while HB-2 exhibits a much lower result corresponding to 52% of HB-3 antioxidant activity. Findings reported in the literature show a wide range of values from 189–263 μmol Trolox/g DW in *O. tenuiflorum* leaves from Thailand [56] up to 1000 μmol Trolox/g extract [58] in studies performed in *Ocimum* spp. leaves from Croatia, with the present results fitting within these ranges.

A different study on *O. tenuiflorum* leaves extract from Thailand reported an IC_50_ value of 98.5 μg extract/mL [49], which would represent higher antioxidant activity than the 27% inhibition achieved using a concentration of 100 μg extract/mL of HB-3 in the present work.

#### 2.4.4. ORAC Evaluation

The ORAC methodology results summarized in Table 4 show again that sample HB-1 has a similar value to HB-3, while HB-2 is the sample with the lowest antioxidant activity. Comparing with the literature, previous studies indicate ORAC results with values ranging between 8.1 and 9.2 µM TE/g FW [45] for leaves of *O. tenuiflorum* cultivated in the USA and 0.8 μmol Trolox/mg extract for *Ocimum* spp. leaves from Croatia [58], lower than the results obtained in the present study.

In sum, the overall comparison of *O. tenuiflorum* leaf extracts from Costa Rica with similar studies in Southeast Asia, Croatia, and the USA shows similarity in most antioxidant results within an appropriate range. The expected variability from the diverse growth environments and from the different harvesting stages, due to the level of polyphenolic content [41,42], is reflected in the antioxidant results. This is linked, as it will be discussed more extensively in the next section, to the extracts component’s chemical structure enabling radical inhibition and thus exerting antioxidant activity [59].

### 2.5. Statistical Analysis

A Pearson correlation analysis (*p* < 0.05) was conducted for the quantification results of phenolic compounds (UPLC), total phenolics (TP), and antioxidant analyses (DPPH, FRAP, TEAC, and ORAC). These results are summarized in Table 5.

**Figure 4:**
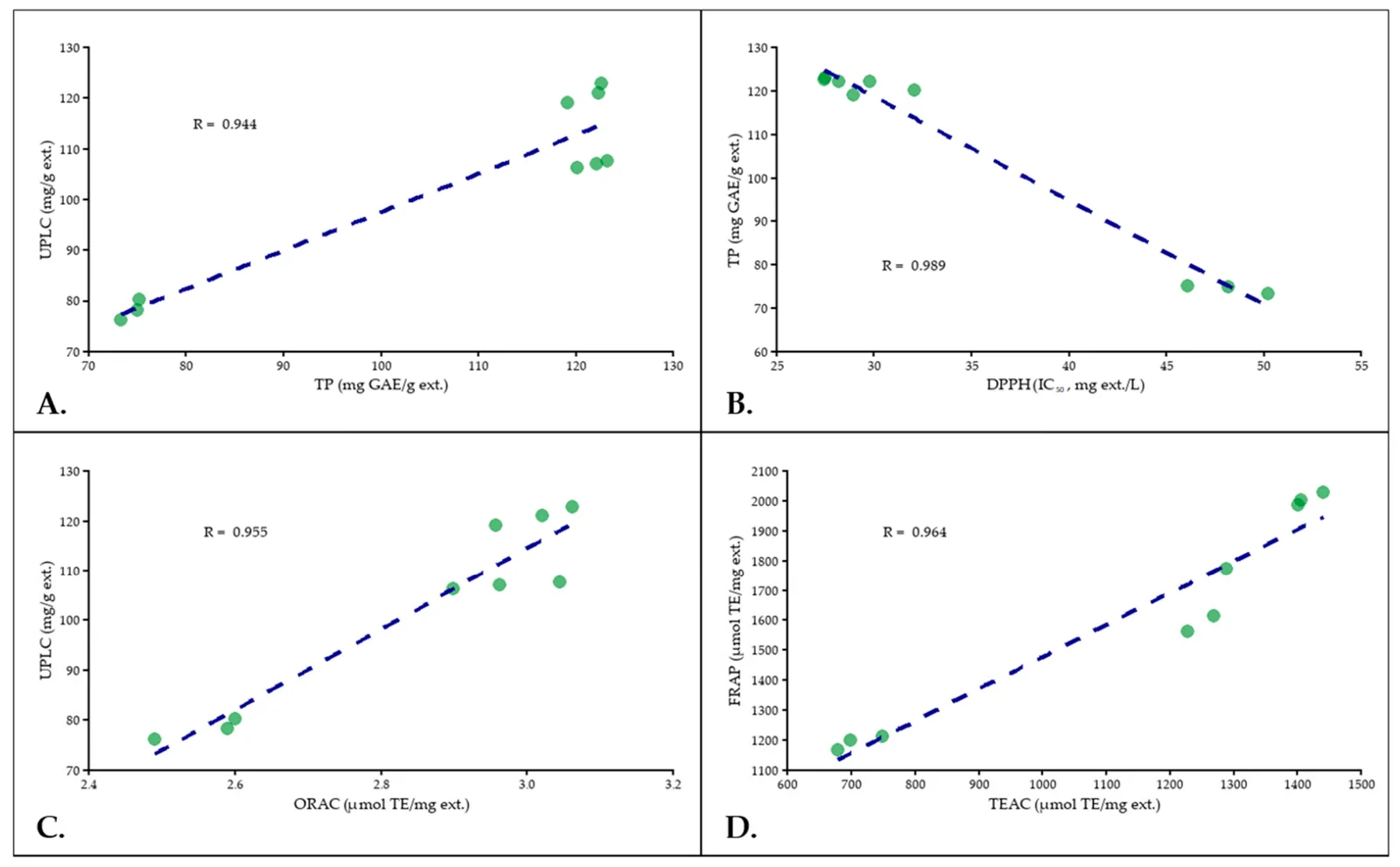
Correlations between polyphenolic content and antioxidant evaluations. (**A**) UPLC-DAD quantification and Total Polyphenols (TP), (**B**) TP and DPPH antioxidant values, (**C**) UPLC-DAD quantification and ORAC antioxidant values, (**D**) FRAP and TEAC antioxidant values.

As shown above in Table 5, results from the quantification of phenolic compounds, UPLC-DAD, and TP exhibit a very strong correlation with antioxidant analyses, namely DPPH, FRAP, ORAC, and TEAC. This is in agreement with previous reports suggesting a direct relationship between polyphenolic compound content and antioxidant activity due to the fact of their structure, including aromatic hydroxyl groups that are able to react with reactive oxidative species (ROS), thus exerting antioxidant effects [52,59]. The negative values in DPPH correlations arise from this method measuring the inhibition concentration, so a better antioxidant capacity implies a lower IC_50_ value. Some of these correlations are shown in Figure 4.

The structure-activity relationship of the main polyphenols present in *O. tenuiflorum* leaves contributes to a better understanding of the correlation with their antioxidant activity. For instance, eugenol **12** has a methylated catechol unit, and rosmarinic acid **10** has two catechol units, one in the caffeoyl moiety and the other in the 2-oxyphenylpropanoyl moiety, which are important substructures for their ability to react with radicals [60,61].

In turn, apigenin and luteolin glucoronides are flavones with a 2-phenylchromen-4-one backbone [24], and the o-dihydroxyl structure in the B-ring has been shown to contribute to effective radical scavenging in luteolin when compared to apigenin, supporting the significance of the catechol ring in the formation of stable radical species [62]. These effects have been demonstrated at the individual compound level and in synergetic effects from these flavonoids with other polyphenolic compounds [24,63].

A Relative Antioxidant Capacity Index (RACI) was calculated for all antioxidant data, namely DPPH, FRAP, ORAC, and TEAC evaluations included in Table 4. RACI consists of a dimensionless quantity of normalized data that allows comparison between different analysis methods [64], as presented here for antioxidant activity with results summarized in Table 6.

This index has been applied successfully in different studies [65,66] demonstrating that a higher value corresponds to the sample with the best biological activity. As can be seen in Table 6, although HB-1 and HB-3 have similar results in some analyses, the RACI value implies that HB-3 is the sample with the best overall performance as an antioxidant, while HB-2 is the one with the least antioxidant activity. These results, as mentioned before, are supported by the difference in harvesting maturity stages of the samples [41,42], with HB-2 corresponding to an earlier stage than HB-1 and HB-3, while the higher value for this last sample can be supported as previously reported by the potential synergistic effects of their polyphenol content [24].

### 2.6. Cytotoxicity Evaluation

**Figure 5:**
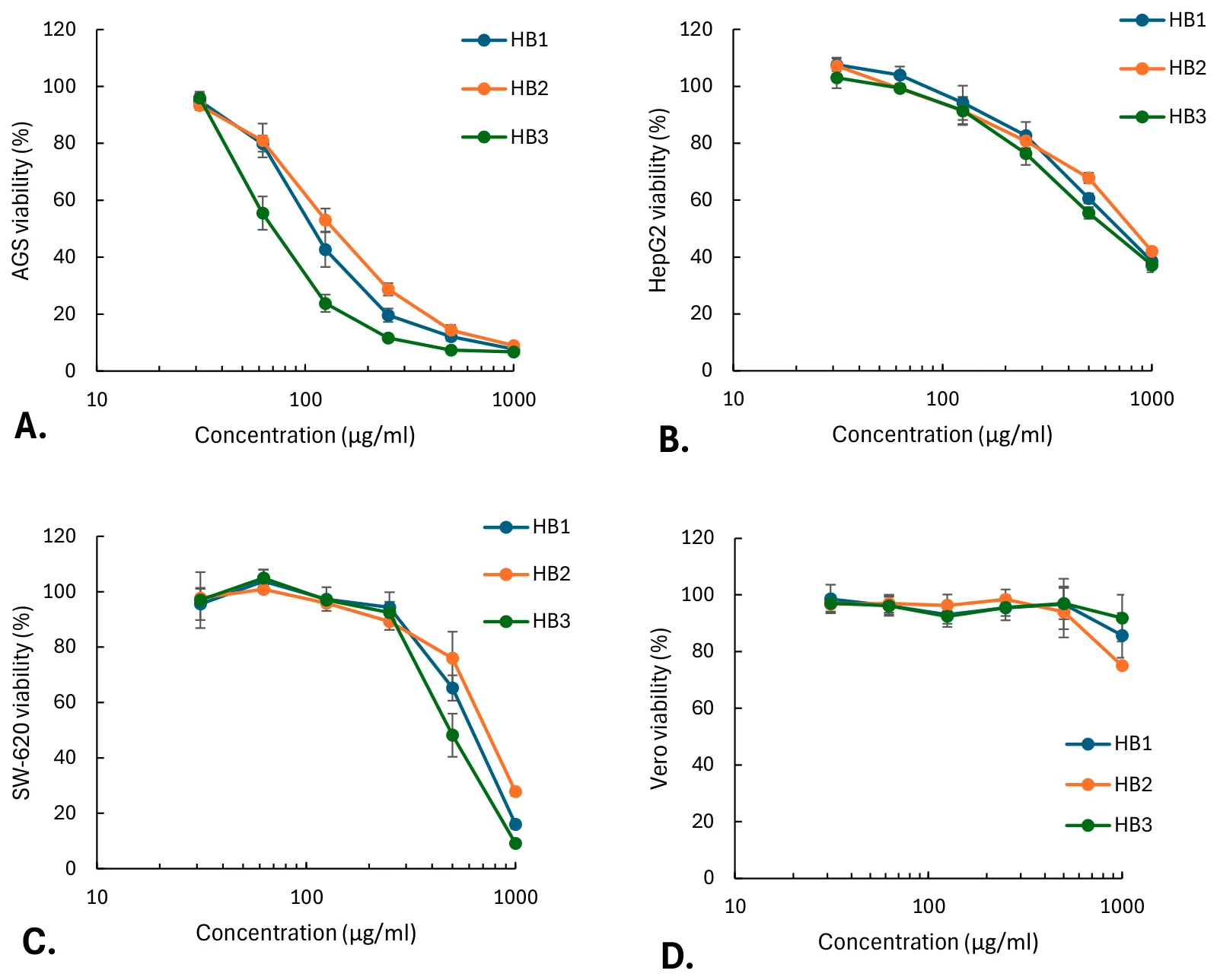
Cytotoxicity dose-response curves of *O. tenuiflorum* extracts in AGS (**A**), HepG2 (**B**), SW-620, (**C**) tumoral cells, and a control non-tumoral cell Vero (**D**). Results are presented as mean ± SD of three independent experiments.

The in vitro evaluation of *O. tenuiflorum* extracts on various adenocarcinoma cell lines revealed differing cytotoxic effects (Table 7, Figure 5). Among the three cancer cell lines studied, namely SW-620 colorectal adenocarcinoma cell lines, HepG2 hepatic adenocarcinoma cell lines, and AGS gastric adenocarcinoma cell lines, all extracts exhibit the most significant cytotoxic effect on AGS cell lines, as indicated by their lower IC_50_ values.

In addition, all extracts demonstrated a high selectivity index for AGS adenocarcinoma cells when compared to their cytotoxic effects in non-tumoral normal Vero cell lines. Among the three evaluated extracts, HB-3 exhibits the highest cytotoxic activity and selectivity index, resulting in an extract with a potential therapeutic interest.

Compared with the scientific literature, there is variability of results on AGS adenocarcinoma gastric cell lines. In fact, a study on the effect of *O. tenuiflorum* leaves from Thailand on AGS cell lines showed an IC_50_ of 163 μg/mL [17] in the upper range obtained in the present study (78–150 μg/mL). Another report on *Ocimum* species (*O. basilicum*) leaves from Iran demonstrated lower antioxidant activity in AGS cells, with IC_50_ values of 3180 μg/mL [67].

Regarding HepG2 hepatoma cells, a study on *O. tenuiflorum* from India reported no cytotoxicity at concentrations of 100 μg/mL [68], aligning with the present results reporting an IC_50_ of 581–764 μg/mL. Finally, in respect to SW620 colorectal adenocarcinoma cells, to the best of our knowledge, no other reports have been found in the literature.

The literature shows reports on other tumoral cell lines with variability in the cytotoxic activity. For instance, *O. tenuiflorum* powdered products commercialized in the USA demonstrated cytotoxic activity in MiaPaCa pancreatic cancer cell lines with IC_50_ values between 113 and 230 μg/mL [19], while leaves from plants cultivated in Korea exhibited an IC_50_ of 200 μg/mL in non-small lung cancer cells (NCI-H460) [18]. Both studies exhibit results in a similar range to the ones obtained in the present study on AGS cancer cells.

Finally, in another study, *O. tenuiflorum* leaves from plants cultivated in India did not exhibit important activity on leukemia (THP-1) [69] and breast cancer (MCF-7) [54] cell lines with IC_50_ values exceeding 1000 μg/mL, similar to the findings in the present report on HepG2 and SW620 adenocarcinoma cell lines.

At the individual level, among the bioactive compounds quantified in the *O. tenuiflorum* extracts, previous studies have reported rosmarinic acid with an IC_50_ of 14 μmol/L against HepG2 liver adenocarcinoma [70] and apigenin with an IC_50_ of 109.3 μmol/L against MCF-7 breast cancer cell lines [71], thus offering promising results, while IC_50_ g = higher values of 300 and 500 μmol/L have been reported for eugenol against HCT-15 and HT-29 colon adenocarcinomas, respectively [72].

These results show the variability dependent on compounds and cell lines due to the complex cytotoxicity patterns mediated through mechanisms, such as enhancing antioxidant activity, altering gene expression, inducing apoptosis, and inhibiting angiogenesis and metastasis [73]. Finally, the results also point to the synergistic effects that can be attained in extracts rich in these polyphenols in the achievement of such mechanisms [24].

Besides the notable cytotoxic activity of *O. tenuiflorum* extracts on AGS cancer cells, these extracts also exhibit a high selectivity index (SI), indicating that extracts are over 6 times more toxic to AGS cancer cells than to the normal Vero cells. HB-3 holding a selectivity index of 12.8, is the sample with the highest SI and therefore, the one with the strongest potential for further studies to gather more evidence in order to accurately determine the cytotoxicity and safety of these extracts, since higher SI values suggest a better outlook towards potential therapeutic applications [74,75]. Further, the higher RACI value of HB-3 antioxidant results aligns with the higher cytotoxicity found for this sample on AGS gastric adenocarcinoma.

## 3. Materials and Methods

### 3.1. Reagents and Solvents

Solvents such as methanol and acetonitrile of HPLC grade were acquired from Baker (Center Valley, PA, USA). Reagents such as quercetin, rosmarinic acid, eugenol, gallic acid, Folin-Ciocalteu reagent, 2,2-diphenyl-1-picrylhidrazyl (DPPH), sodium carbonate, 2,2′-azinobis (3-ethylbenzothiazoline-6-sulphonate) diammonium salt (ABTS), potassium ferricyanide, iron (III) chloride, 2,2′-azobis(2-amidinopropane) dihydrochloride (AAPH), fetal bovine serum, glutamine, penicillin, streptomycin, amphotericin B, and trypsin–ethylenediaminetetraacetic acid (EDTA) were obtained from Sigma-Aldrich (St. Louis, MO, USA). The human gastric adenocarcinoma cell line (AGS), human colorectal adenocarcinoma (SW620), human hepatocellular carcinoma (Hep-G2), and normal monkey epithelial kidney Vero cells were obtained from the American Type Culture Collection (ATCC, Rockville, MD, USA).

### 3.2. Sample Preparation

The aerial parts of holy basil (*O. tenuiflorum*), Rama tulsi cultivar, sold in local markets in Costa Rica, were obtained from producers whose organic plots are located in the southern region, in coordinates 9°19′25.83″ N 83°42′57.82″ W, 9°19′30.45″ N 83°42′52.35″ W, and 9°19′20.65″ N 83°42′41.32″ W, in May 2024. The specimens are kept at the Costa Rican National Herbarium, and the vouchers assigned by Botanist Alonso Quesada correspond to AQ3762, AQ3763, and AQ3764, respectively.

The leaves were manually separated from the stems and were labeled as HB-1, HB-2, and HB-3, respectively. These leaf samples were air-dried at room temperature and then in a Precision 6909 oven (Thermo Scientific™, Walthman, MA, USA) at 40 °C until constant weight was achieved. Once dried, the samples were powdered and preserved in sealed containers at −10 °C.

Extraction was carried out in a Dionex™ ASE™ 150 Accelerated Solvent Extractor (Thermo Scientific™, Walthman, MA, USA), following an adaptation of a previous procedure [52]. Briefly, 5 g of pulverized material was placed in a 34 mL cell using methanol at 40 °C as a solvent under 3 extraction cycles of 10 min each. The extracts were evaporated to dryness in a rotary evaporator Buchi R-100 (Büchi, Flawil, Switzerland) under vacuum. The yields were calculated, and the extracts were frozen stored until further use. Sample solutions for analyses were prepared with 10.0 mg of dry extract dissolved in 10.00 mL of methanol.

### 3.3. UPLC-QTOF Analysis

The UPLC-MS system used to analyze the composition of holy basil extracts consisted of an AQUITY H Class UPLC system with a quaternary pump coupled with a Xevo G2-XS QTOF (Waters, Wilmslow, UK). The analyses followed previous published ESI parameters [76], consisting of 2 kV of capillary voltage, 20 eV in the sampling cone, 150 °C of source temperature, and 10 °C of source offset. Desolvation temperature was set at 450 °C, cone gas flow at 0 L/h, and desolvation gas flow at 900 L/h. Measurement was performed in MSe high-resolution negative mode using an acquisition mass range from 100 m/z to 2000 m/z and a scan rate of 0.5 s, where fragmentation was carried out using Independent Data Acquisition with a collision energy ramp from 20 V to 30 V stored at the high energy function. Instrument calibration was applied in the mass range of the measurement with sodium formate. Lock mass correction was applied directly to the measurement using leucine enkephalin infusion measured each 30 s during the run. The data were analyzed using MassLynx V4.2 software from Waters Corporation (Milford, MA, USA) and MZmine 4 from mzio (Bremen, Germany). Chromatography was carried out on a Luna RP-C18(2) column (150 mm x 4.6 mm i.d. x 4 μm, Phenomenex, Torrance, CA, USA) at 20 °C, using water with 0.1% formic acid (A) and acetonitrile (B) as solvents with a gradient that went from 10% B to 25% B in 20 min, 45% B at 35 min and 70% B 45 min, holding it for 4 min, at a flow rate of 0.5 mL/min.

### 3.4. UPLC-DAD Analysis

For quantification purposes, measurements were performed using a UPLC Ultimate 3000 with Diode Array Detector (DAD) coupled with a simple quadrupole MSQ Plus instrument (ThermoFisher Scientific, San Jose, CA, USA). The analyses followed a modified previously described system [43]. Briefly, a Luna RP-C18 column (150 mm × 4.6 mm i.d. × 4 μm, Phenomenex, Torrance, CA, USA) with a pre-column filter was used. Mobile phases consisted of 0.1% formic acid in water (*v*/*v*) (A) and methanol (B). The gradient started at 10% B and increased to 25% B at 15 min, held until 20 min, then increased again to 30% B at 25 min and 70% B at 45 min, holding it for 2 min. Flow was set at 1.0 mL/min, column temperature at 20 °C and DAD acquisition was set at 278 nm. Calibration curves of quercetin (10–100 ppm), rosmarinic acid (2–250 ppm), and eugenol (10–500 ppm) were prepared. 20 μL of standards or samples were injected, and results were expressed as mg of compound per gram of extract. Flavonoids were quantified with quercetin as standard.

### 3.5. Total Phenolics

The determination was performed through a modified Singleton and Rossi method, employing the Folin–Ciocalteu (FC) reagent, which is composed of a mixture of phosphotungstic and phosphomolybdic acids. As previously reported [52], the assay comprises mixing 0.5 mL of FC reagent with 0.5 mL of the respective extract, adding 10 mL of Na_2_CO_3_ (7.5%), and diluting to 25.00 mL with distilled water. A blank was prepared following the same procedure using 0.5 mL of H_2_O in place of the extract. Samples were kept in the dark at room temperature for 1 h, and afterwards absorbance was measured at 750 nm. The absorbance measurements obtained were extrapolated in a gallic acid calibration curve to obtain Folin–Ciocalteu (FC) reducing capacity results, further expressed as mg gallic acid equivalents (GAE)/g of the extract. Each determination was performed in triplicate.

### 3.6. Antioxidant Activity Evaluation

#### 3.6.1. DPPH Analysis

A DPPH evaluation of samples was conducted as previously reported [76]. In brief, a solution of 2,2-diphenyl-1-picrylhidrazyl (DPPH) (0.25 mM) was prepared in methanol. A volume of 2 mL of this solution was mixed with 0.5 mL of the sample at different concentrations, diluted to 5 mL with methanol, and incubated at 25° C in the dark for 30 min. The DPPH absorbance was measured at 517 nm. Blanks were prepared for each sample, and the DPPH absorbance (*Abs*) was measured at 517 nm. The *Inhibition percentage* was determined as shown:

(1) $$\mathrm{Inhibition}\mathrm{percentage}(\%)=\frac{\left({\mathrm{Abs}}_{\mathrm{blank}}-{\mathrm{Abs}}_{\mathrm{sample}}\right)}{{\mathrm{Abs}}_{\mathrm{blank}}}\times 100$$

The inhibited percentage of the sample was plotted against its concentration to calculate the IC_50_. Each sample was analyzed in three independent assays.

#### 3.6.2. FRAP Analysis

The Ferric Reducing Antioxidant Power (FRAP) was conducted as previously reported [77] with some modifications. In brief, a sample solution was diluted 1:5 in methanol, and an aliquot of 1 mL was mixed with 0.2 mL of HCl 1 mol/L, 1.5 mL of potassium ferricyanide 1%, 0.5 mL of sodium dodecyl sulfate 1%, and 0.5 mL of iron (III) chloride 0.1%. The mixture was completed to 10 mL with distilled water and incubated at room temperature for 30 min prior to measuring the absorbance at 750 nm against a blank and interpolated in a calibration curve of Trolox (50–600 μmol/L). Results are expressed as μmol Trolox equivalent per gram of extract.

#### 3.6.3. TEAC Analysis

In the Trolox Equivalent Antioxidant Capacity (TEAC), a previously reported method [78] was adapted. In brief, an equal volume of ABTS (2,2′-azinobis (3-ethylbenzothiazoline-6-sulphonate) diammonium salt) 7.4 mmol/L and potassium persulfate 2.6 mmol/L were mixed 12 h prior to analysis to form the solution of ABTS*^+^. Before the analysis, an aliquot of this solution was diluted with distilled water to obtain a working solution with an absorbance of approximately 1.0 mAU at 734 nm. A sample solution was diluted 1:10 in methanol, from which an aliquot of 250 μL was mixed with 4750 μL of working solution of ABTS*^+^ and incubated at room temperature in the dark for 2 h. The absorbance was measured at 734 nm against a blank. The inhibition percentage was calculated, and the result was interpolated in a calibration curve of Trolox (5–150 mg/L). Results are expressed as mg Trolox equivalent per gram of extract.

#### 3.6.4. ORAC Analysis

The ORAC (Oxygen Radical Absorbance Capacity) antioxidant activity was determined following a method previously described [79,80] using fluorescein as a fluorescence probe. The reaction was performed in 75 mM phosphate buffer (pH 7.4) at 37 °C. The final assay mixture consisted of AAPH (12 mM), fluorescein (70 nM), and either Trolox (1–8 μM) or the extract at different concentrations. The fluorescence was recorded every minute for 80 min in black 96-well untreated microplates using a Fluoroskan Ascent plate reader (ThermoFisher Scientific, San Jose, CA, USA) with 485-P excitation and 520-P emission filters. Ascent Software version 2.6 (Thermo Labsystems, Helsinki, Finland) was used to measure the fluorescence. Fluorescein was diluted from a stock solution (1.17 mM) in 75 mM phosphate buffer (pH 7.4), while the AAPH and Trolox solutions were freshly prepared before analysis. All the reaction mixtures were prepared in duplicate, and three independent runs were completed for each extract. The fluorescence measurements were normalized, and the area under the fluorescence decay curve (AUC) was calculated. The net AUC corresponding to a sample was calculated as the difference between the sample or Trolox and the blank. The regression equation between the net AUC and sample concentration was calculated. The ORAC value was estimated by dividing the slope (*b*) of the latter equation by the slope of the Trolox line obtained for the same assay.

(2) $$\mathrm{ORAC}=\frac{b_{\mathrm{sample}}}{b_{\mathrm{Trolox}}}$$

The final ORAC values are expressed as the mmol of Trolox equivalents (TE)/g of the sample.

### 3.7. Cytotoxic Evaluation

Three different human cancer cell lines, namely, gastric AGS, colorectal SW-620, and hepatocellular HepG2 cells, as well as monkey normal Vero kidney cells, were used. The analyses followed a methodology previously described [27] with some variations. Minimum essential Eagle’s medium (MEM), holding 10% fetal bovine serum (FBS), served to grow these four cell types, along with amphotericin B (0.25 μg/mL), penicillin (100 IUmL^−1^), glutamine (2 mmol/L), and streptomycin (100 μg/mL). A humidified atmosphere at a temperature of 37 °C with 5% CO_2_ was used. Cells were sub-cultured using a trypsin–EDTA solution for detachment, with a confluence of around 70–80%. Once growth was achieved, a volume of 100 μL of cell suspension (1.5 × 10^5^ cells/mL) was seeded into 96-well plates overnight. Subsequently, 50 μL of sample at different dilutions were added to the seeded cells, and cell culture medium was added to obtain final concentrations from 30 to 1000 μg/mL, then left in incubation for 48 h. A similar procedure was used to prepare control cultures, which were achieved by not adding any extract to the cultured cells.

A cytotoxicity assessment for each sample was achieved through the measurement of the level of reaction of available cells with MTT. After the 48h incubation, the cells’ medium was removed, and PBS (2 × 100 μL) was used to wash the cells. Subsequently, an MTT solution in culture medium (5 mg/mL) was added (100 μL), and the mixture was left for incubation at 37 °C for 2 h. Afterwards, 100 μL of 95% ethanol was added to dissolve the formazan crystals, and the plates were put in a microplate reader to measure the absorbance at a wavelength of 570 nm. Dose–response curves were established for each sample and concentration, and, finally, the cell viability reduction by 50% (IC_50_) was calculated from these curves. The selectivity index (*SI*) was calculated as the relation between the IC_50_ value obtained in a cancer cell line in respect to the IC_50_ in the normal Vero cell lines.

(3) $$\mathrm{SI}=\frac{{\mathrm{IC}}_{50\;\mathrm{Vero}}}{{\mathrm{IC}}_{50\mathrm{Cancer}\;\mathrm{cell}\;\mathrm{line}}\;}$$

### 3.8. Statistical Analysis

One-way analysis of variance (ANOVA), followed by Tukey’s post hoc test, was applied to phenolic quantification, antioxidant, and cytotoxicity values, and differences were considered significant at *p* < 0.05. Pearson correlation analyses were performed (*p* < 0.05) among all four antioxidant activities, quantified metabolites, and total polyphenols. A R program version 4.4.2 with RStudio version 2024.12.0+467 (Posit Software, Boston, MA, USA) was used.

For total phenolics (TP) and antioxidant analyses (DPPH, FRAP, ORAC, and TEAC), the Relative Antioxidant Capacity Index (RACI) was calculated as the average of standard scores. For each analysis, standard scores (*SS*) were calculated as:

(4) $$\mathrm{SS}=\frac{x-\mu }{\sigma }$$

With *x* being the raw data for the analysis, *μ* is the mean among samples and *σ* the respective standard deviation. For DPPH, as expressed as IC_50_, data were converted to 1/IC_50_ for RACI calculations [34,35].

## 4. Conclusions

The UPLC-QTOF characterization of *O. tenuiflorum* leaf extracts allowed the identification of 12 polyphenolic compounds, including seven glycosylated flavonoids, two coumaroyl alcohol derivatives, rosmarinic acid, eugenol, and salvianolic acid E. The quantification by UPLC-DAD revealed HB-1 and HB-3 samples to hold similar higher total polyphenolic content in respect to HB-2, attributed to a lower harvesting maturity stage for this last sample. The different antioxidant analyses also showed that HB-1 and HB-3 exhibited higher activity, and HB-2 had consistently the lower values. The results displayed a remarkably high correlation between UPLC-DAD polyphenolic quantification, TP, and antioxidant activities, in agreement with previous studies on the polyphenolic structure-activity relationship. In addition, all samples exhibited important cytotoxic activity against AGS adenocarcinoma cell lines, with a selectivity index greater than 6. Sample HB-3 is the sample with the most promising potential for further studies, holding the best RACI value representing the greater overall antioxidant activity, exhibiting the best cytotoxic results against AGS cancer cells, and having a selectivity index of 12 in respect to Vero normal cells, indicating a synergistic effect of its polyphenol composition. This work offers a thorough study of *O. tenuiflorum* leaf extracts and constitutes the first study in samples from Costa Rica, showing promising antioxidant and antitumoral activities. However, further studies are needed to assess potential limitations such as safety and efficacy as well as to gather clinical data to accurately evaluate their potential as natural therapeutic agents.

## Figures and Tables

**Table 1:** Main identified compounds in *O. tenuiflorum* through UPLC QTOF analysis.

No.	Compound	Rt (min)	[M − H]^−^	Formula	Fragments
1	Luteolin-7-*O*-glucoside	18.75	447.0926	C_21_H_19_O_11_	285
2	Vitexin	20.28	431.0980	C_21_H_19_O_10_	283, 311, 341
3	Kaempferol-7-*O*-glucoside	21.65	447.0926	C_21_H_19_O_11_	285, 284
4	Luteolin-7-*O*-glucuronide	23.09	461.0721	C_21_H_17_O_12_	285
5	Kaempferol-3-*O*-glucoside	24.60	447.0926	C_21_H_19_O_11_	284, 255
6	Apigenin-7-*O*-glucoside	25.26	431.0980	C_21_H_19_O_10_	269, 268
7	*p*-Coumaryl alcohol 4-*O*-glucoside	25.38	311.1129	C_15_H_19_O_7_	149
8	Salvianolic acid E	25.59	717.1447	C_36_H_29_O_16_	519, 475, 339
9	Apigenin-7-*O*-glucuronide	26.79	445.0767	C_21_H_17_O_11_	269
10	Rosmarinic acid	27.73	359.0766	C_18_H_15_O_8_	179, 161, 135, 133
11	*p*-Coumaryl alcohol 4-*O*-acetylglucoside	29.91	353.1234	C_17_H_21_O_8_	293, 149
12	Eugenol	31.05	163.0755	C_10_H_11_O_2_	148

**Table 2:** Quantification of phenolic compounds in *O. tenuiflorum* samples by UPLC-DAD.

Compound	Rt (min)	HB-1	HB-2	HB-3
		(a) Concentration in mg/g extract ^1,2^		
Luteolin-7-*O*-glucoronide	15.66	9.49 ± 0.30 ^a,&^	12.91 ± 0.01 ^a,#^	10.36 ± 0.07 ^a,†^
Apigenin-7-*O*-glucoronide	18.55	9.32 ± 0.24 ^a,&^	12.25 ± 0.02 ^a,#^	10.73 ± 0.45 ^a,†^
Rosmarinic acid	19.46	15.75 ± 0.51 ^b,&^	5.38 ± 0.88 ^b,#^	5.43 ± 0.06 ^b,#^
Eugenol	34.76	86.49 ± 1.87 ^c,&^	47.74 ± 2.03 ^c,#^	80.52 ± 1.39 ^c,†^
Total		**121.05 ± 1.84 ^&^**	**78.28 ± 2.92 ^#^**	**107.04 ± 0.95 ^†^**
		**(b) Concentration in mg/g dry weight ^1,3^**		
Luteolin-7-*O*-glucoronide	15.66	0.481 ± 0.015 ^a,&^	0.733 ± 0.001 ^a,#^	0.569 ± 0.004 ^a,†^
Apigenin-7-*O*-glucoronide	18.55	0.473 ± 0.012 ^a,&^	0.695 ± 0.001 ^a,#^	0.590 ± 0.025 ^a,†^
Rosmarinic acid	19.46	0.799 ± 0.026 ^b,&^	0.306 ± 0.035 ^b,#^	0.298 ± 0.003 ^b,#^
Eugenol	34.76	4.388 ± 0.095 ^c,&^	2.710 ± 0.082 ^c,#^	4.425 ± 0.077 ^c,&^
Total		**6.141 ± 0.093 ^&^**	**4.443 ± 0.117 ^#^**	**5.883 ± 0.052 ^†^**

^1^ In sections (a) and (b), values are expressed as average ± standard deviation (SD). ^2^ In section (a), different superscript letters in the same column, and different symbols in the same row indicate significant differences (*p* < 0.05) using one-way analysis of variance (ANOVA) with a post-hoc Tukey as statistical analysis test. ^3^ In section (b), different superscript letters in the same column, and different symbols in the same row indicate significant differences (*p* < 0.05) using one-way analysis of variance (ANOVA) with a post-hoc Tukey as statistical analysis test.

**Table 3:** Total Phenolic Content (TP) of *O. tenuiflorum* samples.

Sample	TP	TP
	(mg GAE ^1^/g Extract) ^2,3^	(mg GAE/g DW ^4^)
HB-1	122.5 ± 0.3 ^a^	6.215 ± 0.013 ^a^
HB-2	74.5 ± 1.1 ^b^	4.229 ± 0.061 ^b^
HB-3	121.8 ± 1.5 ^a^	6.696 ± 0.084 ^c^

^1^ GAE: gallic acid equivalents. ^2^ Values are expressed as average ± standard deviation (SD). ^3^ Different superscript letters in the same column indicate significant differences (*p* < 0.05) using one-way analysis of variance (ANOVA) with a post-hoc Tukey as statistical analysis test. ^4^ DW: dry weight.

**Table 4:** Antioxidant activities of *O. tenuiflorum* samples.

Sample	DPPH IC_50_	FRAP	ORAC	TEAC
	**(a) Values per extract weight ^1,2^**			
	**mg extract/L**	**μmol TE ^3^/g extract**	**μmol TE/mg extract**	**μmol TE/g extract**
HB-1	28.18 ± 0.75 ^a^	1649.6 ± 109.5 ^a^	3.013 ± 0.053 ^a^	1261.1 ± 30.8 ^a^
HB-2	48.18 ± 2.08 ^b^	1192.8 ± 22.4 ^b^	2.563 ± 0.061 ^b^	708.7 ± 36.3 ^b^
HB-3	29.79 ± 2.30 ^a^	2006.5 ± 21.5 ^c^	2.969 ± 0.074 ^a^	1415.8 ± 21.5 ^c^
	**(b) Values per dry sample weight (DW) ^1,4^**			
	**mg DW/L**	**μmol TE/g DW**	**μmol TE/mg DW**	**μmol TE/g DW**
HB-1	555.4 ± 14.8 ^a^	83.7 ± 5.6 ^a^	0.1529 ± 0.0027 ^a^	64.0 ± 1.6 ^a^
HB-2	848.9 ± 36.6 ^b^	67.7 ± 1.3 ^b^	0.1455 ± 0.0035 ^b^	40.2 ± 2.1 ^b^
HB-3	542.0 ± 41.8 ^a^	110.3 ± 1.2 ^c^	0.1631 ± 0.0041 ^a^	77.8 ± 1.2 ^c^

^1^ Values in section (a) and (b) are expressed as average ± standard deviation (SD). ^2^ In section (a), different superscript letters in the same column indicate significant differences (*p* < 0.05) using one-way analysis of variance (ANOVA) with a post-hoc Tukey as statistical analysis test. ^3^ TE: Trolox equivalents. ^4^ In section (b), different superscript letters in the same column indicate significant differences (*p* < 0.05) using one-way analysis of variance (ANOVA) with a post-hoc Tukey as statistical analysis test.

**Table 5:** Pearson correlation values ^1^ obtained for polyphenolics UPLC quantification, TP, and antioxidant evaluations in samples of *O. tenuiflorum*.

	UPLC	TP	DPPH	FRAP	TEAC
TP	0.944				
DPPH	−0.962	−0.989			
FRAP	0.709	0.896	−0.855		
TEAC	0.860	0.979	−0.956	0.964	
ORAC	0.955	0.976	−0.993	0.846	0.940

^1^ *p* < 0.05, for a confidence level of 95%.

**Table 6:** RACI ^1^ values obtained for samples of *O. tenuiflorum*, based on results per mass of extract and mass of dried weight.

Sample	HB-1 ^2^	HB-2 ^2^	HB-3 ^2^
RACI (extract)	0.48 ± 0.26	−1.12 ± 0.05	0.65 ± 0.21
RACI (DW ^3^)	0.16 ± 0.30	−1.04 ± 0.11	0.88 ± 0.19

^1^ RACI: Relative Antioxidant Capacity Index; ^2^ RACI value ± standard deviation (SD). ^3^ DW: Dry weight.

**Table 7:** Cytotoxicity of *O. tenuiflorum* extracts on gastric (AGS), hepatic (HepG2), and colorectal (SW620) adenocarcinoma cell lines, as well as non-tumoral cell (Vero).

Samples	AGS		HepG2		SW620		Vero
	IC_50_ ^1,2,3^	SI ^4^	IC_50_	SI	IC_50_	SI	IC_50_
HB-1	128 ± 8 ^a^	7.8 ± 0.5	673 ± 42 ^ab^	1.5 ± 0.1	674 ± 62 ^ab^	1.5 ± 0.1	>1000
HB-2	150 ± 10 ^a^	6.7 ± 0.4	764 ± 15 ^a^	1.31 ± 0.03	784 ± 49 ^a^	1.3 ± 0.1	>1000
HB-3	78 ± 5 ^b^	12.8 ± 0.7	581 ± 55 ^b^	1.7 ± 0.2	562 ± 46 ^b^	1.8 ± 0.1	>1000

^1^ IC_50_ in μg extract/mL. ^2^ Values are expressed as the mean ± standard deviation (SD). ^3^ Different superscript letters in the same column indicate significant differences (*p* < 0.05) ^4^ SI: Selectivity Index values expressed as mean ± standard deviation (SD).

## Data Availability

The original contributions presented in this study are included in the article/Supplementary Materials. Further inquiries can be directed to the corresponding author.

## Supplementary Materials

The following supporting information can be downloaded at: https://www.mdpi.com/article/10.3390/molecules31193546/s1, Figure S1: Extracted ion chromatograms (XIC) from UPLC-QTOF-ESI analysis for the identified compounds in *O. tenuiflorum*; Figure S2: UPLC-DAD chromatograms for (a) HB-1, (b) HB-2, and (c) HB-3 extracts.
