# Supplementary material for: Polyphenolic HRMS Characterization, Contents, Antioxidant Activity and Cytotoxic Effects of Costa Rican Ocimum tenuiflorum L. Extracts
Source: Molecules. 2026 Oct 4;31(19):3546. doi: 10.3390/molecules31193546 (PMC13666767; doi:10.3390/molecules31193546)
Supplement: Supplementary file 1 [file molecules-31-03546-s001.zip › molecules-4535836-supplementary.pdf]

SUPPLEMENTARY MATERIALS

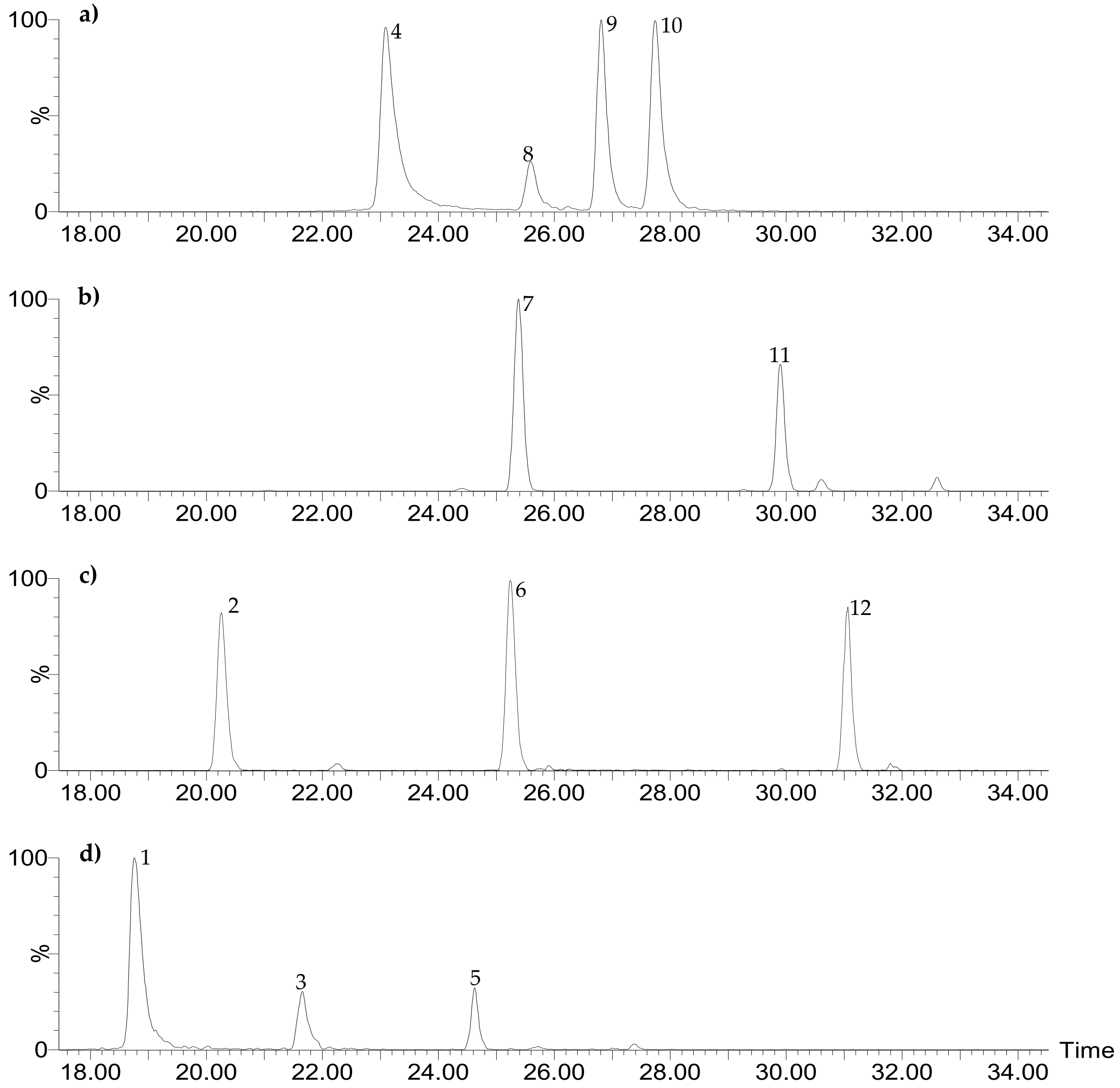

Figure S1. Extracted ion chromatograms (XIC) from UPLC-QTOF-ESI analysis for the identified compounds in *O. tenuiflorum*. a) Peaks **4**, **8**, **9** and **10**; b) peaks **7** and **11**; c) peaks **2**, **6** and **12**; d) peaks **1**, **3** and **5**.

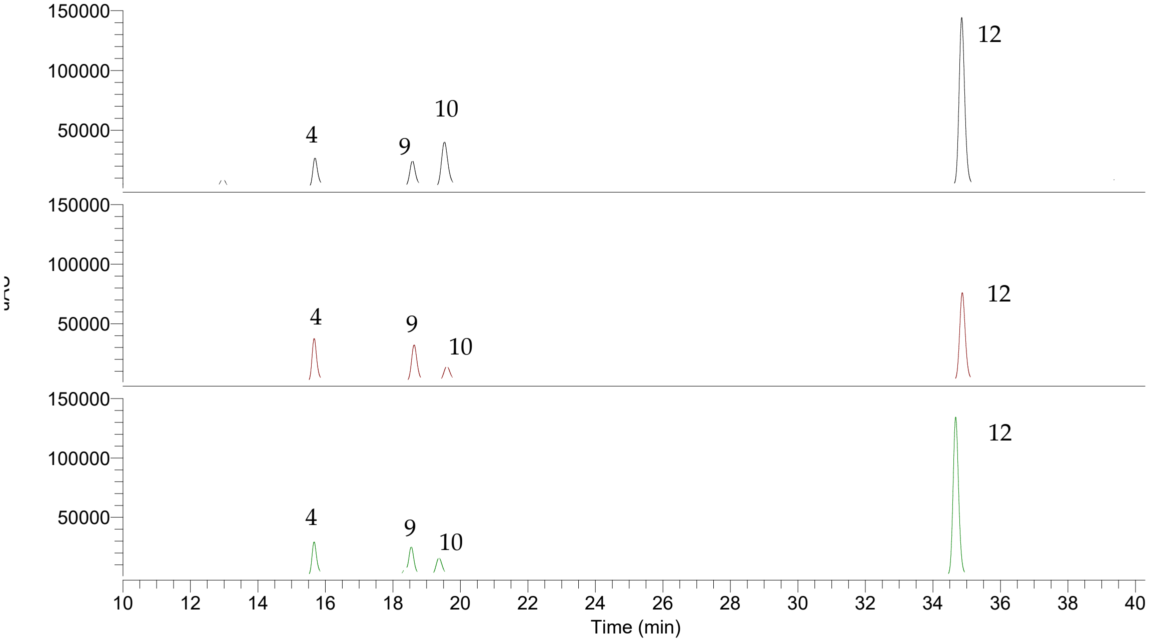

Figure S2. UPLC-DAD chromatograms for a) HB-1, b) HB-2, and c) HB-3 extracts. Peak **4**: luteolin-7-O-glucoronide, Peak **9**: apigenin-7-O-glucoronide, Peak **10**: rosmarinic acid, and Peak **12**: eugenol.
